# *DMD* Open‐access Variant Explorer (DOVE): A scalable, open‐access, web‐based tool to aid in clinical interpretation of genetic variants in the *DMD* gene

**DOI:** 10.1002/mgg3.510

**Published:** 2018-11-18

**Authors:** Mitchell Bailey, Nicole Miller

**Affiliations:** ^1^ BioMarin Pharmaceutical Inc. Novato California

**Keywords:** bioinformatics, Duchenne, medical genetics, molecular diagnostics, precision medicine

## Abstract

**Background:**

Duchenne muscular dystrophy (Duchenne) is caused by pathogenic variants in the *DMD* gene. Antisense oligonucleotides (AONs) are one emerging precision medicine treatment for Duchenne. *DMD* molecular genetic testing results guide precision‐therapy molecular eligibility, requiring healthcare providers to perform analyses currently uncommon in clinical laboratory and medical practices. Clear *DMD* variant notation and interpretation are key components of clinical care with the availability of precision medicine.

**Methods:**

The *DMD* Open‐access Variant Explorer (DOVE) is a web‐based aid for *DMD* variant interpretation which additionally reports variant‐specific predicted molecular eligibility for therapy. DOVE was developed in Python and adapted to the Django Web framework, integrates existing open‐access tools, and does not rely on previous variant report/classification.

**Results:**

DOVE [www.dmd.nl/DOVE] interprets colloquial and HGMD inputs of *DMD* variants to output HGMD variant nomenclature, theoretical molecular eligibility for therapy, and any predicted deleterious molecular consequences of therapy. DOVE relies on holistic in silico prediction of molecular eligibility for therapy in lieu of reference to an empirically defined, “variant‐eligible” list. Examples illustrate the advantage and necessity for holistic variant interpretation.

**Conclusion:**

DOVE may prove useful for variant interpretation both at patient‐level and in large‐scale programs such as newborn screening and has broad application in concept to molecular genetic test result interpretation.

## 1 INTRODUCTION

**Figure 1:**
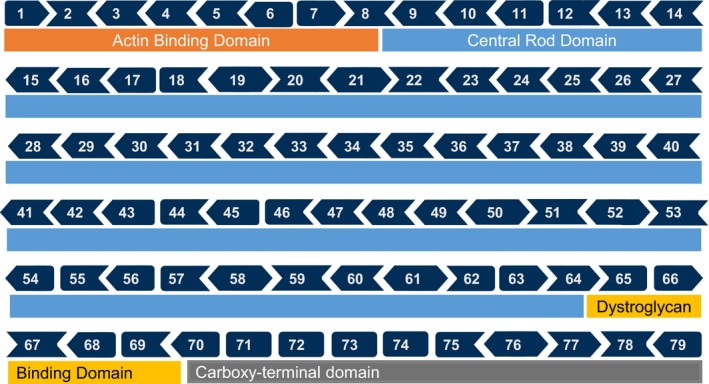
Exons of the *DMD *gene.*The majority of deletions in the *DMD* gene occur in the highly repetitive Central Rod Domain. Deletions disrupting the reading frame generally give rise to the severe, Duchenne, phenotype. In‐frame deletions in this region are associated with a milder phenotype associated with Becker. *This figure correctly displays the exon 75–78 junctions. Previous literature reports a different reading frame

Individuals with Duchenne muscular dystrophy (Duchenne) lack functional dystrophin protein expression due to pathogenic variants in the *DMD* (OMIM 300377) gene located on the X‐chromosome (Xp21). This results in childhood‐onset progressive muscle degeneration and death in late adolescence to early adulthood. In muscle tissue, dystrophin's structural role as a cytoskeletal stabilization protein protects muscle fibers against contraction‐induced damage. In the absence of dystrophin, the supportive link between the cytoskeleton and extracellular matrix is destabilized leading to easily damaged muscle fibers. Dystrophin‐deficient, damaged muscle tissue is replaced by adipose and fibrotic connective tissue (Kharraz, Guerra, Pessina, Serrano, & Muñoz‐Cánoves, 2014). Duchenne thus originates from a molecular pathology that occurs due to activities of daily living: force‐induced cellular damage in skeletal muscle results in a combination of muscle fiber replacement, impaired vascular function, ischemia, and metabolic stress. As a progressive muscular dystrophy, impaired lower body muscle function is followed by loss of ambulation, loss of upper limb function, decreased respiratory function, cardiomyopathy, and death by the second to third decade of life (Kieny et al., 2013). Becker muscular dystrophy (Becker) typically presents with later onset and slower disease progression in comparison with Duchenne. While Becker also results from pathogenic variants in *DMD*, the genetic variants found in Becker‐diagnosed individuals are generally distinguishable from those found in Duchenne. Most Duchenne‐causing mutations in *DMD* are either large deletions or large duplications in the gene, removing one or multiple exons and disrupting the translational open reading frame (Beggs, Koenig, Boyce, & Kunkel, 1990; Bladen et al., 2015). Duchenne‐causing mutations yield trace to no detectable dystrophin production (Muntoni, Torelli, & Ferlini, 2003). The majority of Becker cases are also caused by large deletions in *DMD* but are distinguished by preservation of the translational open reading frame, resulting in production of shorter dystrophin protein with both *N*‐ and C‐terminal binding domains intact, preserving a degree of dystrophin function (Muntoni et al., 2003). In both Duchenne and Becker, most large mutations occur in the highly repetitive central rod domain (Figure 1, Supporting information Supplement S1).

The advent of molecular therapy for Duchenne underscores the necessity to accurately describe the pathogenic variant in *DMD* with respect not only to muscular dystrophy diagnosis, but also to accurately offer genetic counseling in context of potential molecular eligibility for precision medicine‐based treatment of disease. Here, eligibility for therapy relies on an accurate clinical diagnosis and essentially on an accurate interpretation of the *DMD* variant. The molecular techniques employed for characterization of the *DMD* variant have been recently well‐reviewed (Aartsma‐Rus, Ginjaar, & Bushby, 2016). Briefly, because a vast majority of dystrophy‐causing mutations in *DMD* are the result of large deletions in the gene, multiple independent ligation‐dependent probe amplification is the most practical first test offered for diagnosis of Duchenne. Nominally, a validated *DMD* next generation sequencing (NGS) approach is the most accurate and comprehensive approach, although this may not be the most cost‐effective approach for some molecular laboratories (Wei et al., 2014). To date, there are two precision medicines on the market for Duchenne and at least three others in development (ClinicalTrials.gov, 2015: NCT02500381; PTC Therapeutics International Ltd., 2014; Sarepta Therapeutics Inc., 2016). Currently, there is not a centralized approach to aid determination of molecular eligibility for existing and in‐development precision medicines for Duchenne. While several *DMD* gene “open reading frame checkers” exist, none to‐date adequately address the evolving molecular approach to diagnosis and treatment of Duchenne. An option specifically for determining exon‐skip eligibility for large deletions in the *DMD* gene is using the “exon reading frame shape approach” (Figure 1). This visual aid is effective for assessing reading frame corrections, but is prone to human error and is distinct from resources used for interpretation of other variant types in the *DMD* gene. Here, we describe a novel bioinformatics tool for use in streamlining and simplifying interpretation and reporting of *DMD* molecular testing results for genetic counseling purposes and to aid in molecular eligibility determination.

## 2 *DMD* OPEN‐ACCESS VARIANT EXPLORER (DOVE) TOOL

### 2.1 Overview

**Figure 2:**
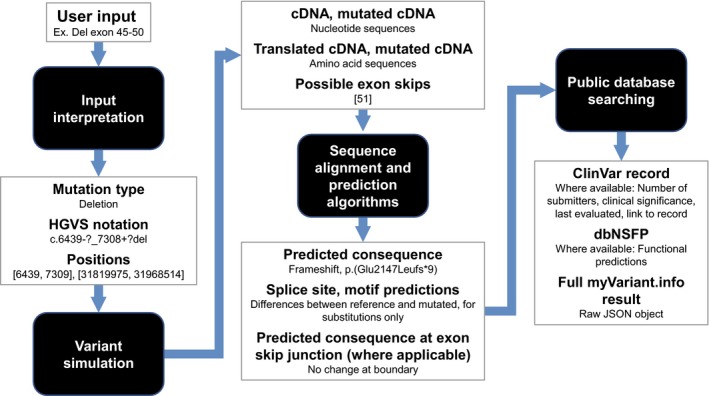
Scheme of DOVE. Rounded black boxes indicate a module. Bold black font indicates module input/output, regular black font provides more detail or an example using “Del exon 45–50” the user input, where applicable

**Figure 3:**
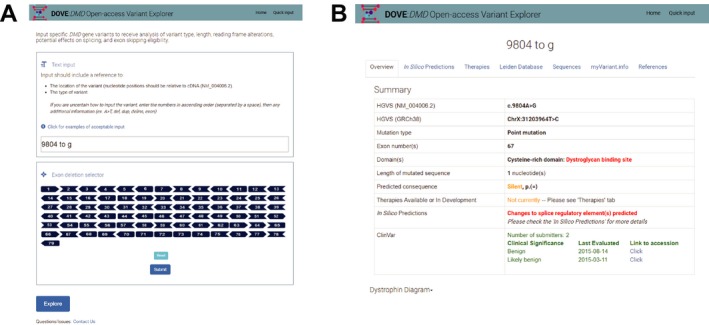
DOVE interface. Example of input “9804 t to g” given. (a) Text input and exon deletion selector page. (b) Results overview page. Additional pages include In silico predictions, Theoretical therapeutic eligibility and the Leiden Open Variation Database search window

The automated, web‐based tool, "*DMD* Open‐access Variant Explorer" (DOVE), was created to interpret any variant in the *DMD* gene. This approach combines several freely available web tools and scripts to interpret different aspects of the variation (e.g., impact on amino acid sequence or splicing) in one step, reducing the need for numerous searches and manual organization of results. The tool can be broken into four functional modules: (1) input interpretation, (2) variant simulation, (3) sequence alignment and prediction algorithms, and (4) public database searching. The modules, along with the general input and output of each, are included in Figure 2. Each module roughly represents one step of analysis which would generally be performed separately with a distinct online tool (e.g., portions of the input interpretation and sequence alignment modules could be completed with the Mutalyzer tool available at Leiden University Medical Center; Wildeman, van Ophuizen, Dunnen, & Taschner, 2008). This approach can be applied both to observed and novel variants encountered. The user interface has been designed to allow for input of both Human Genome Variation Society (HGVS)‐recommended nomenclature (den Dunnen et al., 2016), “verbal” notation (e.g., deletion of exon 50, dup of exon 2), as well as “visual input” for exon deletions using the exon schematic (Figure 3a, Supporting information Supplement S2). Both input types will be output to the user in HGVS‐compliant notation (den Dunnen et al., 2016).

### 2.2 Input interpretation module

Due to heterogeneity in nomenclature for variants, particularly copy number variants (CNVs), the input interpretation module was developed to process text‐based user input. This module distinguishes between various input formats: “exon notation” (e.g., “deletion of exon 45”), “verbal notation” (e.g., “4806 a to t”), or HGVS‐recommended coding sequence notation (e.g., “c.4806A>T”). A list of key character combinations was created to determine the type of variant from the text provided (Supporting information Supplement [Link], [Link]). Regardless of input format, the input interpretation module provides a structured output for use in the variant simulation module: variant type, inserted or changed nucleotides (if applicable), starting position, and ending position. If all of the required data cannot be determined from user input, the interpretation module will redirect the user to triage pages to specifically ask for the missing information (e.g., for a user input of “del 45,” the triage page will ask whether the user is referring to deletion of *exon* 45 or deletion of *nucleotide* 45). While DOVE uses a specific *DMD* reference sequence (NM_004006.2), this module could easily be applied to any reference sequence for any gene.

By removing stringency on user input, the user input module increases accessibility to variable users, with variable familiarity with HGVS variant nomenclature. Interpretation of user input and display of HGVS‐compliant variant notation aids in dissemination of variant notation best practices. In addition, by providing the user with HGVS‐compliant variant nomenclature, DOVE facilitates accurate searching of public databases, both automated (DOVE) and manually by the user.

### 2.3 Variant simulation module

The DOVE variant simulation uses the *DMD* Dp427m transcript (NM_004006.2) as a reference to simulate the mutated sequence, based on the output of the input interpretation module. For large deletions and duplications involving entire exons, this module also simulates removal of the left, right, two left, two right, or left and right flanking exons. Only exon‐skip simulated sequences which are theoretically exon‐skip amenable will be stored. Theoretical exon skipping eligibility is defined by restoration of the reading frame (i.e., whether the deletion length, including the length of the exon or exons to be skipped, or the duplication length less the length of the exons removed, is divisible by three). After variant simulation, this module translates each of the coding sequences produced and stores the resulting, mutated amino acid sequences.

### 2.4 Sequence alignment and prediction algorithms module

A translated version of the *DMD* Dp427m transcript is used to identify changes to the amino acid sequence introduced by the variant. Each amino acid change is stored until a termination codon is reached in the mutated sequence. Additionally, exon‐skip simulated sequences are then translated to assess whether an amino acid substitution or premature termination codon has been created at the simulated in‐frame exon–exon junction. For single‐nucleotide variations (SNVs), splice site and splicing motif algorithms are used to score both the wild‐type and mutated sequences. For splice site changes, the MaxEnt algorithm is implemented using Perl wrappers made available by Yeo and Burge (2004). Using the MaxEnt algorithm, if there is a difference in score between reference sequence or simulated sequence and either sequence returns a score of 3 or greater in the variant location, a report will be provided to the user. Splicing motifs are scored or identified with ESEFinder matrices, Rescue‐ESE and Fas‐ESS hexamers (Cartegni, Wang, Zhu, Zhang, & Krainer, 2003; Fairbrother, Yeh, Sharp, & Burge, 2002; Smith et al., 2006; Wang et al., 2004). This process assumes that all positions surrounding the SNV match the reference sequence (NM_004006.2), thus introducing a limitation to variant‐by‐variant analysis. Nonsynonymous SNVs are assessed with functional predictions retrieved from dbNSFP using a static file including all positions in the *DMD* gene (Liu, Wu, Li, & Boerwinkle, 2016). Functions were developed to retrieve functional prediction information using myVariant.info, but were less reliable in retrieving available information; the retrieved results are available for download by the user (Xin et al., 2016).

### 2.5 Public database searching module

The variant's ClinVar record is retrieved via the myVariant.info API using HGVS‐compliant variant notation and gene symbol. The clinical classification of the variant, date last evaluated, and a link to the accession for the corresponding variant is formatted for display. All other myVariant.info data are made available to download in a designated tab of the results page.

Relevant information from the Leiden Open Variation Database (LOVD) is displayed in an embedded window. In this inline frame, users can access information at LOVD associated with previous reports of the variant.

### 2.6 Output

A results overview page is provided once the variant has been analyzed by the program (Figure 3b, Supporting information Supplement S2). The overview includes information related to the position of the variant (relative to coding sequence, genomic sequence, exon number, and domain), the length of the variant (relative to the coding sequence), the type of variant, the predicted consequence on the amino acid sequence, and summaries for the “potential therapies,” “in silico predictions,” and ClinVar reports. All variant descriptions are provided in HGVS‐recommended notation. A schematic depiction of the dystrophin protein domains, overlaid with exon and amino acid positions, is provided in the overview tab for quick reference. Text in the overview is color‐coded based on general impact of that type of information. For example, “frameshift,” “actin binding domain,” and “nonsense” will appear in red text; “missense,” and “silent” will appear in yellow; and “possibly therapies available” will appear in green. Information with indeterminate importance appears in black. If a frameshift or nonsense variant is input by the user, a notification of potential therapies is provided. Potential therapies include premature termination codon read‐through therapy and any theoretically possible exon skipping therapy.

## 3 DISCUSSION

In addition to providing proper notation of molecular genetic test results, this tool provides information regarding the exon and domain location of the variant, and the amino acid change (in HGVS notation). In silico splicing motif and splice site algorithms (ESEFinder, Rescue‐ESE, Fas‐ESS hexamers, and MaxEnt) as well as functional predictions available from dbNSFP are applied and reported in a simplified format (Cartegni et al., 2003; Liu et al., 2016; Smith et al., 2006; Wang et al., 2004; Yeo & Burge, 2004). The ClinVar and LOVD databases are searched and summaries are provided (Aartsma‐Rus, Deutekom, Fokkema, Ommen, & Dunnen, 2006; Landrum et al., 2015). For large deletions in the gene, the tool provides all possible exon skips to correct the disruptive reading frame in addition to any codon changes predicted to occur at the newly created exon–exon boundary of the cDNA. This approach has shown increased utility over static lists of skip amenable deletions (Supporting information Supplement [Link], [Link]); private or novel mutations may not appear on such lists. Lists, while simple to interface with, also must be maintained uniquely for every possible exon‐skip. Additionally, currently available static lists do not report introduction of nucleotide and/or amino acid changes at newly created boundaries post‐exon‐skip, leaving the potential for ineffective interventions such as in the event a stop codon is introduced at a newly created junction.

**Figure 4:**
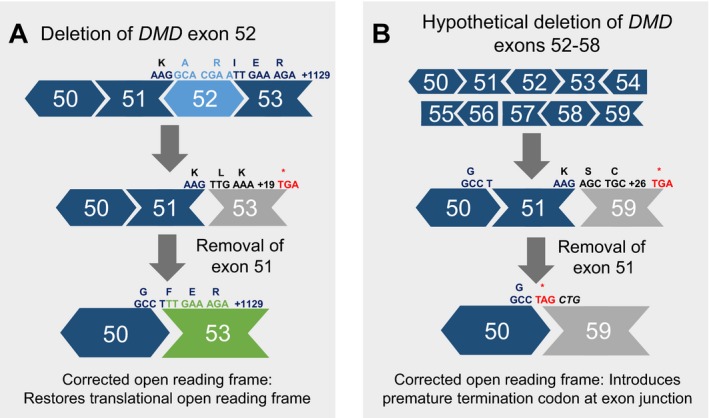
CNVs in the *DMD* gene frequently cause a frameshift and little to no functional dystrophin production. (a) Removal of one or more exons (e.g., by AON exon skipping therapy), the reading frame may be corrected to allow for production of functional dystrophin. (b) When an exon is skipped, there is the potential for the introduction of a new amino acid, or premature termination codon

A theoretical deletion of exon 52–58 in the *DMD* gene (not present in LOVD) causes a frameshift to result in subsequent loss of the C‐terminus of dystrophin. This frameshift is theoretically exon 51‐skip amenable: removing exon 51 would restore the reading frame. However, removing exon 51 introduces a codon change at the boundary of exons 50 and 59, introducing a premature termination codon (Figure 4). While hypothetical, this illustrates the need for holistic variant interpretation and points to an advantage to sequence‐level analysis alongside reading frame assessment when considering exon skipping therapy.

**Figure 5:**
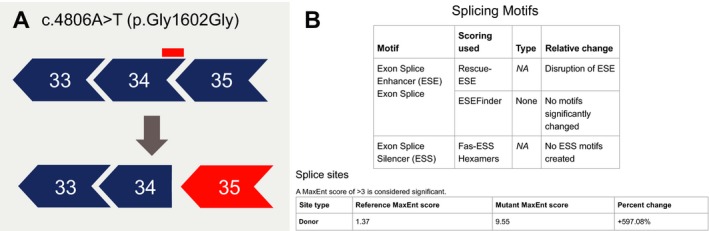
DOVE assesses all SNVs for changes to ESE, ESS, or splice sites. (a) The silent, mid‐exonic *DMD* variant, c.4806A>T, has been determined to cause a partial loss of exon 34 due to aberrant splicing and subsequent frameshift. (b) DOVE results page for NM_004006.2(*DMD*):c.4806A>T: Predicted disruption of an ESE consensus site and simultaneous creation a novel ESS consensus site

Analysis of splice site and splicing motif alterations yielded potential utility to identify splice‐altering SNVs. The mid‐exonic, silent *DMD* variant (c.4806A>T) is predicted to disrupt a wild‐type ESE motif while substantially increasing the MaxEnt donor splice site score of a cryptic site (Figure 5). This prediction is consistent with laboratory findings from RNA analysis performed on this allele by Emory Genetics Laboratory, revealing a frameshift due to partial loss of exon 34 caused by aberrant splicing. While in silico predictions are not generally accepted as a means to define splice‐altering variants, this type of result could indicate cause for pursuing further analysis (e.g., RNA analysis) and further emphasizes the benefit of holistic variant analysis.

While interpretation of *DMD* variants as Duchenne‐ versus Becker‐associated is not always straightforward, most often pathogenic *DMD* variants can reasonably be classified as either Becker or Duchenne. With the advent of molecular targeted therapies, such as AONs for Duchenne, a more detailed understanding of molecular genetic test results is required. Improper or incomplete interpretation can lead to misguided treatment or management of disease, as illustrated by the splicing example (c.4806A>T) and the newly created termination codon example (deletion of exon 52–58 with exon 51 skipping; Figure 4). While some diagnostic laboratories may include molecular eligibility for targeted therapies in test reports, this is not commonly done particularly for investigational compounds which could impact awareness of the patient and clinician of clinical trial eligibility. Understandably, *previously *diagnosed Duchenne individuals also may be unaware of eligibility for new molecular targeted therapy.

Interpreting a variant, particularly a novel variant, can be time‐consuming. While there are several free tools available online to gather evidence of clinical significance, the burden to navigate several websites with different interfaces and input requirements is high. Some of these websites require accurate HGVS notation, which can be difficult to determine in some cases and may require additional searches (e.g., for exon positions).

In addition to proper understanding of sequence variants, unambiguous variant description is crucial. Without uniform variant description, there is the possibility for miscommunication of results and inefficient aggregation of information for variants encountered elsewhere. There are currently tools available to address this concern (e.g., Mutalyzer; Wildeman et al., 2008). The functionality of DOVE relies on standard, structured information for exploring different aspects of the variation. This structured information can easily be extended to adhere to the recommended notation. While the module implemented here is not immediately extensible to other genes like tools available elsewhere, it provides additional value for subsequent database and literature searches for variants in the *DMD* gene.

We demonstrate that this comprehension can be facilitated using publicly available tools and broadly applicable algorithms. This approach could also easily be extended to any gene using automated sequence retrieval (e.g., from the UCSC DAS server [Kent et al., 2002]). In the future, molecular therapy eligibility tools such as DOVE could be used in large‐scale applications, such as newborn screening.

The utility of DOVE was tested by a group of Duchenne and molecular genetic testing experts in Leiden at and following an expert meeting sponsored by BioMarin Pharmaceutical Inc. At this meeting, variants encountered in clinical practice were tested and output validated against comparable, established data sources (e.g., NCBI ClinVar, Human Splice Finder, manual review of reading frame shift). After this meeting, a mix of variants were tested against established resources, including various types of variants, both recorded and never encountered but theoretically possible. All variants tested yielded expected results. Although rigorous software validation was not performed, the resource was further evaluated after transfer to Leiden by LOVD curators and users.

## 4 LIMITATIONS

DOVE assesses intronic variants based only on their impact on splice site and splicing motifs. Notably, if multiple variants are provided, DOVE will interpret the first variant only.

## 5 SUMMARY

In summary, we have developed a novel bioinformatics tool integrating multiple existing databases, algorithms, and tools available to facilitate efficient analysis of variants in the *DMD* gene. This tool is freely available at the Leiden Open Variation Database (www.dmd.nl/DOVE). The methods used to develop this tool can be replicated in other genes and, potentially, a central platform.

## DISCLOSURES

Mitch Bailey, MS, was an employee of BioMarin Pharmaceutical Inc. at the time of this research and Nicole Miller, PhD, is an employee of BioMarin Pharmaceutical Inc.
