# Supplementary material for: DMD Open‐access Variant Explorer (DOVE): A scalable, open‐access, web‐based tool to aid in clinical interpretation of genetic variants in the DMD gene
Source: Mol Genet Genomic Med. 2018 Nov 18;7(1):e00510. doi: 10.1002/mgg3.510 (PMC6382494; doi:10.1002/mgg3.510)
Supplement: Supplementary file 1 [file MGG3-7-na-s001.pdf]

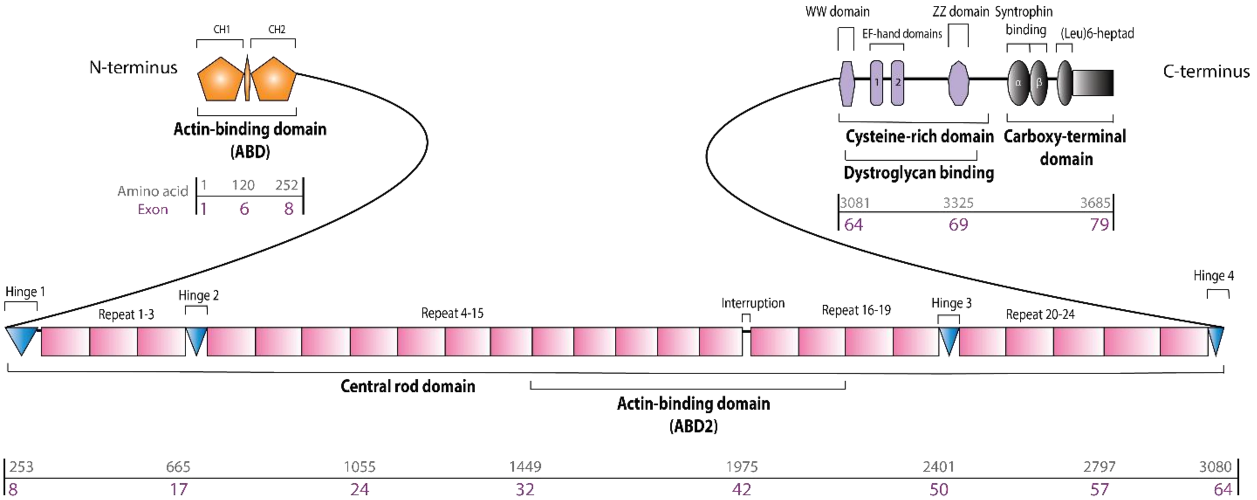


**Figure 1** Domains of the dystrophin protein overlaid with exon boundaries (NM_004006.2) and amino acid positions.
