# Supplementary material for: DMD Open‐access Variant Explorer (DOVE): A scalable, open‐access, web‐based tool to aid in clinical interpretation of genetic variants in the DMD gene
Source: Mol Genet Genomic Med. 2018 Nov 18;7(1):e00510. doi: 10.1002/mgg3.510 (PMC6382494; doi:10.1002/mgg3.510)
Supplement: Supplementary file 2 [file MGG3-7-na-s002.pdf]

Supplement S2. Examples of the search screen and results tabs within the DOVE interface.

Input page

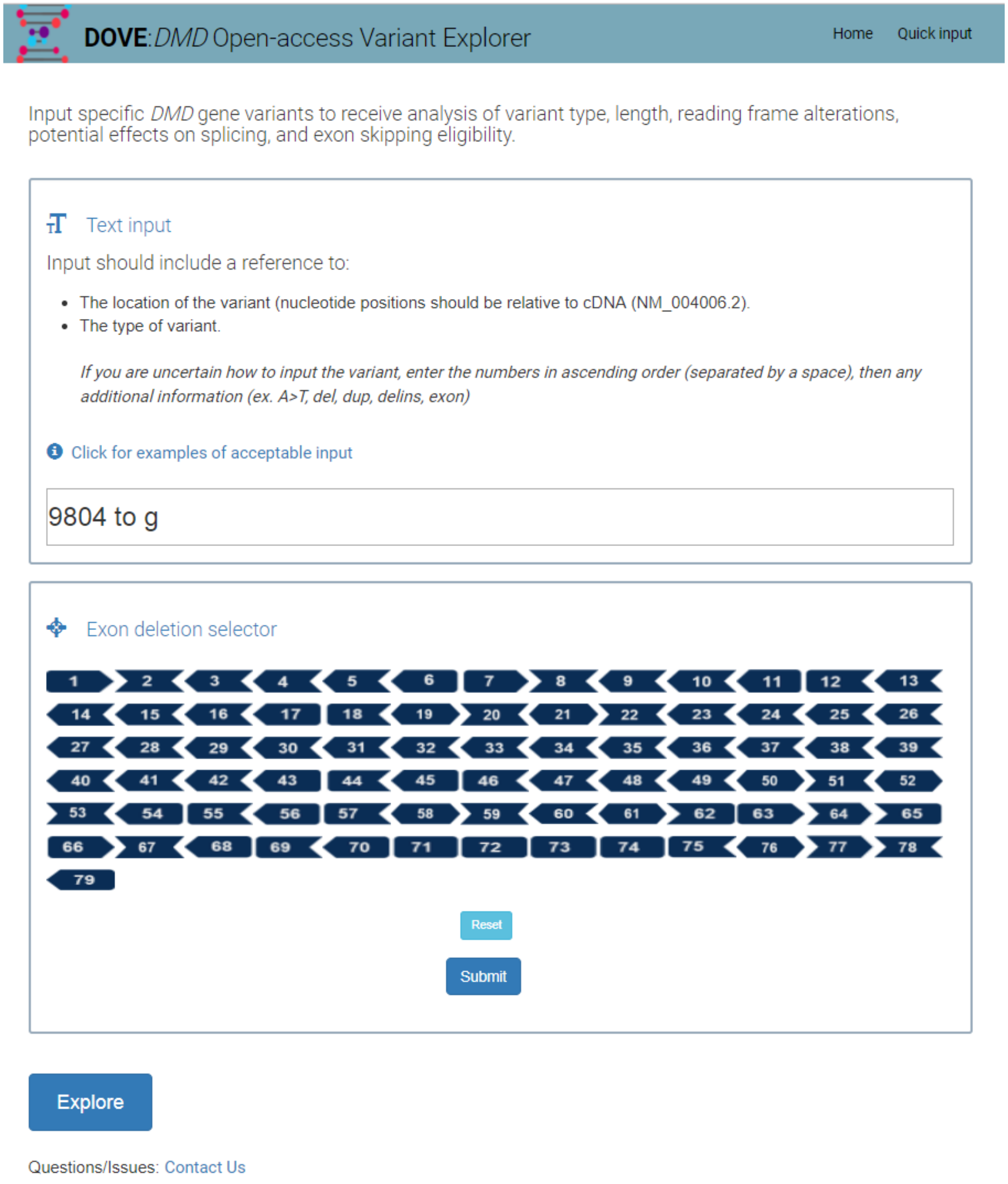

Results summary page

DOVE: *DMD* Open-access Variant Explorer — Home — Quick input

9804 to g

Overview | *In Silico* Predictions | Therapies | Leiden Database | Sequences | myVariant.info | References

Summary

HGVS (NM_004006.2)	**c.9804A>G**
HGVS (GRCh38)	**ChrX:31203964T>C**
Mutation type	**Point mutation**
Exon number(s)	**67**
Domain(s)	**Cysteine-rich domain: Dystroglycan binding site**
Length of mutated sequence	**1** nucleotide(s)
Predicted consequence	**Silent, p.(=)**
Therapies Available or In Development	Not currently -- Please see 'Therapies' tab
In Silico Predictions	**Changes to splice regulatory element(s) predicted**<br>*Please check the 'In Silico Predictions' for more details*
ClinVar	Number of submitters: 2<br>**Clinical Significance** / **Last Evaluated** / **Link to accession**<br>Benign / 2015-08-14 / Click<br>Likely benign / 2015-03-11 / Click

Dystrophin Diagram▾

In silico predictions page

DOVE: *DMD* Open-access Variant Explorer — Home — Quick input

9804 to g

Overview | *In Silico* Predictions | Therapies | Leiden Database | Sequences | myVariant.info | References

Functional Predictions from dbNSFPv3.0

**Negative functional prediction vote (by algorithm): N/A**	
SIFT	Not missense
PolyPhen	Not missense
LRT	Not missense
MutationTaster	Not missense
MutationAssessor	Not missense
FATHMM	Not missense
PROVEAN	Not missense
MetaSVM	Not missense

Splicing Motifs

**Motif**	**Scoring used**	**Type**	**Relative change**
Exon Splice Enhancer (ESE)	Rescue-ESE	*NA*	No change predicted
	ESEFinder	None	No motifs significantly changed
Exon Splice Silencer (ESS)	Fas-ESS Hexamers	*NA*	Mutation creates a novel ESS motif

Splice sites

A MaxEnt score of >3 is considered significant.

**Site type**	**Reference MaxEnt score**	**Mutant MaxEnt score**	**Percent change**
**No changes predicted**			

Targeted therapies prediction page

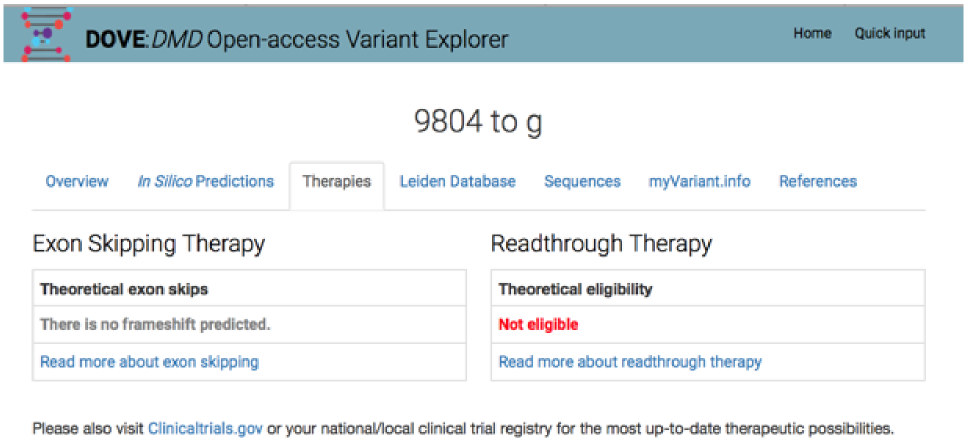

LOVD search page

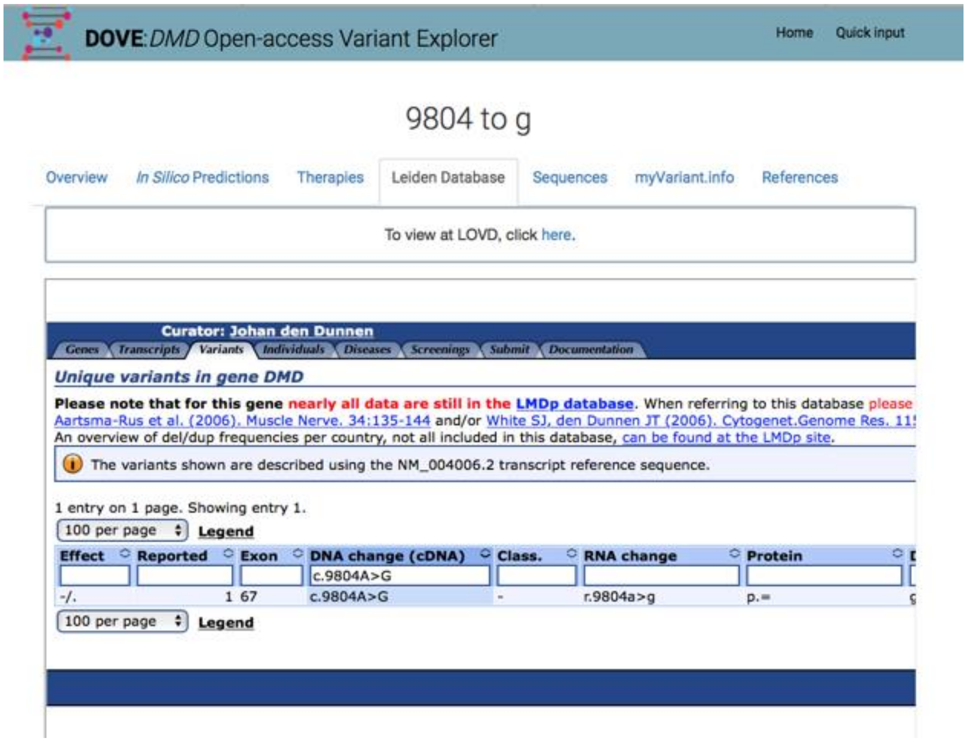

Simulated sequence page

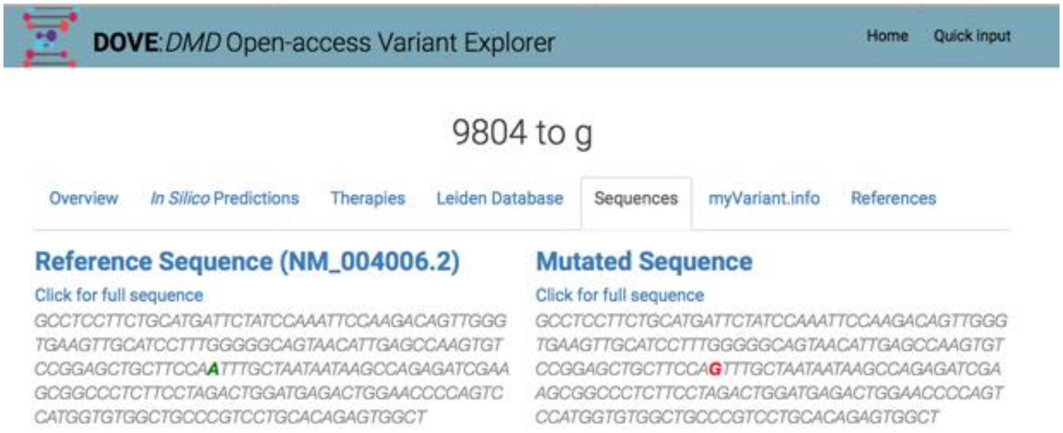

myVariant.info data page

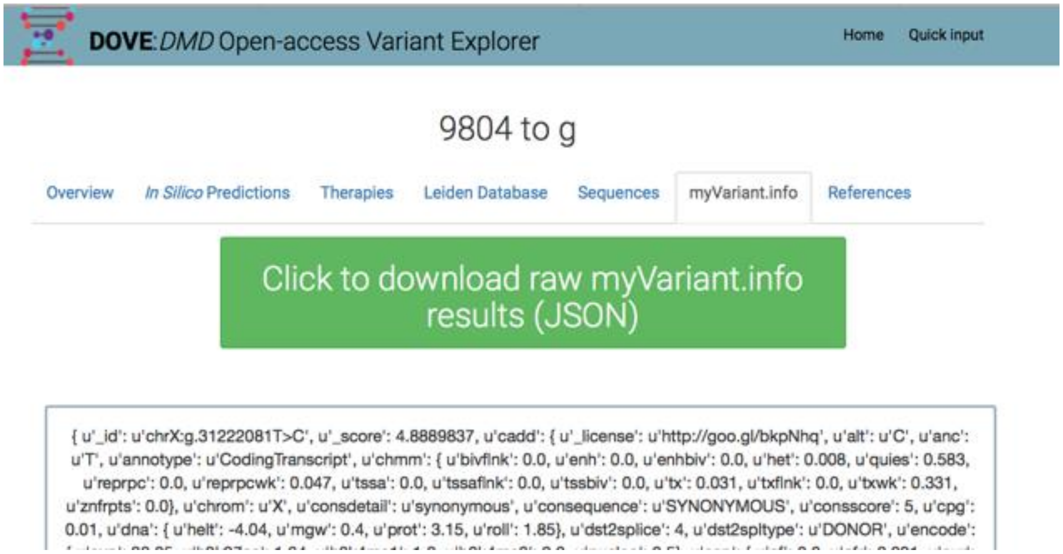

References used for tool development

9804 to g

Overview | *In Silico* Predictions | Therapies | Leiden Database | Sequences | myVariant.info | References

This is not an exhaustive list of work done on exon skipping in the DMD gene.
If a paper/group has been missed, please Contact Us.

Exon Skipping Therapy

- Aartsma-Rus A. Antisense-mediated modulation of splicing: therapeutic implications for Duchenne muscular dystrophy. RNA Biol. 2010 Jul-Aug;7(4):453-61.Epub 2010 Jul 1. Review. PubMed PMID: 20523110.
- Aartsma-Rus, A., Fokkema, I., Verschuuren, J., Ginjaar, I., van Deutekom, J., van Ommen, G. J., & den Dunnen, J. T. (2009). Theoretic applicability of antisense-mediated exon skipping for Duchenne muscular dystrophy mutations. Human mutation, 30(3), 293-299. PubMed PMID: 19156838.
- Arechavala-Gomeza V, Graham IR, Popplewell LJ, Adams AM, Aartsma-Rus A, Kinali M, Morgan JE, van Deutekom JC, Wilton SD, Dickson G, Muntoni F. Comparative analysis of antisense oligonucleotide sequences for targeted skipping of exon 51 during dystrophin pre-mRNA splicing in human muscle. Hum Gene Ther. 2007 Sep;18(9):798-810. PubMed PMID: 17767400.
- Greer KL, Lochmiler H, Flanigan K, Fletcher S, Wilton SD. Targeted exon skipping to correct exon duplications in the dystrophin gene. Mol Ther Nucleic Acids. 2014 Mar 18;3:e155. doi: 10.1038/mtna.2014.8. PubMed PMID: 24643206; PubMed Central PMCID: PMC3982197.
- Matsuo M, Takeshima Y, Nishio H. Contributions of Japanese patients to development of antisense therapy for DMD. Brain Dev. 2015 Jun 18. pii:S0387-7604(15)00108-4. doi: 10.1016/j.braindev.2015.05.014. [Epub ahead of print] Review. PubMed PMID: 26094594.
- Van Deutekom JC, Bremmer-Bout M, Janson AA, Ginjaar IB, Baas F, den Dunnen JT, van Ommen GJ. Antisense-induced exon skipping restores dystrophin expression in DMD patient derived muscle cells. Hum Mol Genet. 2001 Jul 15;10(15):1547-54. PubMed PMID: 11468272.
- Wilton SD, Fall AM, Harding PL, McClorey G, Coleman C, Fletcher S. Antisense oligonucleotide-induced exon skipping across the human dystrophin gene transcript. Mol Ther. 2007 Jul;15(7):1288-96. Epub 2007 Feb 6. PubMed PMID: 17285139.
- Wilton SD, Lloyd F, Carville K, Fletcher S, Honeyman K, Agrawal S, Kole R. Specific removal of the nonsense mutation from the mdx dystrophin mRNA using antisense oligonucleotides. Neuromuscul Disord. 1999 Jul;9(5):330-8. PubMed PMID: 10407856.

Read-through Therapy

- Aurino, S., & Nigro, V. (2006). Readthrough strategies for stop codons in Duchenne muscular dystrophy. Acta myol, 25(1), 5-12. PubMed PMID: 17039975.
- Cossu, G., & Sampaolesi, M. (2007). New therapies for Duchenne muscular dystrophy: challenges, prospects and clinical trials. Trends in molecular medicine, 13(12), 520-526. PubMed PMID: 17983835.
- Finkel, R. S. (2010). Read-through strategies for suppression of nonsense mutations in Duchenne/Becker muscular dystrophy: aminoglycosides and ataluren (PTC124). Journal of child neurology. PubMed PMID: 20519671.
- Hoffman, E. P., Bronson, A., Levin, A. A., Takeda, S. I., Yokota, T., Baudy, A. R., & Connor, E. M. (2011). Restoring dystrophin expression in duchenne muscular dystrophy muscle: progress in exon skipping and stop codon read through. The American journal of pathology, 179(1), 12-22. PubMed PMID: 21703390.
- Kayali, R., Ku, J. M., Khitrov, G., Jung, M. E., Prikhodko, O., & Bertoni, C. (2012). Read-through compound 13 restores dystrophin expression and improves muscle function in the mdx mouse model for Duchenne muscular dystrophy. Human molecular genetics, 21(18), 4007-4020. PubMed PMID: 22692682.
- Malik, V., Rodino-Klapac, L. R., Viollet, L., Wall, C., King, W., Al-Dahhak, R., ... & Mendell, J. R. (2010). Gentamicin-induced readthrough of stop codons in Duchenne muscular dystrophy. Annals of neurology, 67(6), 771-780. PubMed PMID: 20517938.
- Malik, V., Rodino-Klapac, L. R., Viollet, L., & Mendell, J. R. (2010). Aminoglycoside-induced mutation suppression (stop codon readthrough) as a therapeutic strategy for Duchenne muscular dystrophy. Therapeutic advances in neurological disorders, 3(6), 379-389. PubMed PMID: 21179598.
- Zingman, L. V., Park, S., Olson, T. M., Alekseev, A. E., & Terzic, A. (2007). Aminoglycoside-induced translational read-through in disease: overcoming nonsense mutations by pharmacogenetic therapy. Clinical Pharmacology & Therapeutics, 81(1), 99-103. PubMed PMID: 17186006.

Splice Sites & Motifs

- Cartegni L., Wang J., Zhu Z., Zhang M. Q., Krainer A. R.; 2003. ESEfinder: a web resource to identify exonic splicing enhancers. Nucleic Acid Research, 2003, 31(13):3568-3571. PubMed PMID: 12824367.
- Fairbrother WG, Yeh RF, Sharp PA, Burge CB. Predictive identification of exonic splicing enhancers in human genes. Science. 2002 Aug 9;297(5583):1007-13. PubMed PMID: 12114529.
- Gene Yeo and Christopher B. Burge. 2003. Maximum entropy modeling of short sequence motifs with applications to RNA splicing signals. In Proceedings of the seventh annual international conference on Research in computational molecular biology (RECOMB '03), Martin Vingron, Sorin Istrail, Pavel Pevzner, and Michael Waterman (Eds.). ACM, New York, NY, USA, 322-331. PubMed PMID: 15285897.
- Smith, P. J., Zhang, C., Wang, J. Chew, S. L., Zhang, M. Q. and Krainer, A. R. 2006. An increased specificity score matrix for the prediction of SF2/ASF-specific exonic splicing enhancers. Hum. Mol. Genet. 15(16):2490-2508. PubMed PMID: 16825284.
- Wang Z, Rolish ME, Yeo G, Tung V, Mawson M, Burge CB. Systematic identification and analysis of exonic splicing silencers. Cell. 2004 Dec 17;119(6):831-45. PubMed PMID: 15607979.

Data & Sequences

- Aartsma-Rus et al. (2006). Entries in the Leiden Duchenne muscular dystrophy mutation database: an overview of mutation types and paradoxical cases that confirm the reading-frame rule. Muscle Nerve. 34:135-144. PubMed PMID: 16770791.
- Landrum MJ, Lee JM, Riley GR, Jang W, Rubinstein WS, Church DM, Maglott DR. ClinVar: public archive of relationships among sequence variation and human phenotype. Nucleic Acids Res. 2014 Jan 1;42(1):D980-5. doi: 10.1093/nar/gkt1113. PubMed PMID: 24234437.
- Liu X, Wu C, Li C and Boerwinkle E. 2015. dbNSFP v3.0: A One-Stop Database of Functional Predictions and Annotations for Human Non-synonymous and Splice Site SNVs. Human Mutation. PubMed PMID: 26555599.
- White SJ, den Dunnen JT (2006). Copy number variation in the genome; the human DMD gene as an example. Cytogenet.Genome Res. 115: 240-246. PubMed PMID: 17124406.
- Homo sapiens dystrophin (DMD), transcript variant Dp427m, mRNA. RefSeq Accession: NM_004006.2.
- Homo sapiens dystrophin (DMD), RefSeqGene (LRG_199) on chromosome X. RefSeq AFccession: NG_012232.1.
