# Supplementary material for: DMD Open‐access Variant Explorer (DOVE): A scalable, open‐access, web‐based tool to aid in clinical interpretation of genetic variants in the DMD gene
Source: Mol Genet Genomic Med. 2018 Nov 18;7(1):e00510. doi: 10.1002/mgg3.510 (PMC6382494; doi:10.1002/mgg3.510)
Supplement: Supplementary file 3 [file MGG3-7-na-s003.pdf]

Supplement S3. Key character combinations. Character combinations with specificity to a mutation type are used in determining the type of mutation in the user's text input.

Unique character combination (case insensitive)	Instruction
**del**	Set Variant type to deletion
**dup**	Set Variant type to duplication
**del** AND **ins**	Set Variant type to deletion/insertion
**>** **OR** **point** **OR** **trans**	Set Variant type to point change
**x**	Interpret numbers as exon numbers *(Default is to interpret as nucleotides)*
(**+** OR **-**) NOT **x**	Store numbers immediately following symbols as intron references
