# Supplementary material for: DMD Open‐access Variant Explorer (DOVE): A scalable, open‐access, web‐based tool to aid in clinical interpretation of genetic variants in the DMD gene
Source: Mol Genet Genomic Med. 2018 Nov 18;7(1):e00510. doi: 10.1002/mgg3.510 (PMC6382494; doi:10.1002/mgg3.510)
Supplement: Supplementary file 4 [file MGG3-7-na-s004.pdf]

Supplement S4. LOVD-reported *DMD* deletions: reading frame predicted to be corrected by exon 51 skipping. Light blue shading indicates deletions with at least one entry in LOVD.

Theoretical exon 51 skip-amenable deletion*	Number of patients in LOVD**	Theoretical exon 51 skip-amenable deletion*	Number of patients in LOVD**
6-50	0	34-50	0
10-50	0	35-50	0
11-50	0	36-50	0
13-50	1	37-50	0
14-50	0	38-50	0
15-50	0	39-50	0
16-50	0	40-50	1
19-50	2	41-50	0
21-50	0	42-50	0
23-50	0	43-50	2
24-50	0	45-50	220
25-50	0	47-50	31
26-50	0	48-50	212
27-50	0	49-50	151
28-50	0	50	100
29-50	1	52	116
30-50	1	52-58	0
31-50	0	52-61	0
32-50	0	52-63	1
33-50	0		

* Theoretical defined as a deletion where the reading frame is predicted to be corrected with removal of exon 51 according to RefSeq NM_004006.2

**LOVD: Leiden Open Variation Database, entry corresponds to one reported case of Duchenne or Becker muscular dystrophy (accession date March 31, 2017)
